# Persistence of Antibodies against Middle East Respiratory Syndrome Coronavirus

**DOI:** 10.3201/eid2210.160706

**Published:** 2016-10

**Authors:** Daniel C. Payne, Ibrahim Iblan, Brian Rha, Sultan Alqasrawi, Aktham Haddadin, Mohannad Al Nsour, Tarek Alsanouri, Sami Sheikh Ali, Jennifer Harcourt, Congrong Miao, Azaibi Tamin, Susan I. Gerber, Lia M. Haynes, Mohammad Mousa Al Abdallat

**Affiliations:** Centers for Disease Control and Prevention, Atlanta, Georgia, USA (D.C. Payne, B. Rha, J. Harcourt, C. Miao, A. Tamin, S.I. Gerber, L.M. Haynes);; Jordan Field Epidemiology Training Program, Amman, Jordan (I. Iblan);; Jordan Ministry of Health, Amman (S. Alqasraw, A. Haddadin, T. Alsanouri, S. Sheikh Ali, M.M. Al Abdallat);; Eastern Mediterranean Public Health Network, Amman (M. Al Nsour, T. Alsanouri)

**Keywords:** MERS-CoV, Middle East respiratory syndrome, serology, antibody, novel coronavirus, severe acute respiratory illness, viruses, Jordan, respiratory infections

## Abstract

To determine how long antibodies against Middle East respiratory syndrome coronavirus persist, we measured long-term antibody responses among persons serologically positive or indeterminate after a 2012 outbreak in Jordan. Antibodies, including neutralizing antibodies, were detectable in 6 (86%) of 7 persons for at least 34 months after the outbreak.

Middle East respiratory syndrome coronavirus (MERS-CoV) causes acute respiratory illness, which can progress rapidly to respiratory failure and death in ≈40% of persons with laboratory-confirmed cases. The first known cases of MERS-CoV occurred during an outbreak of severe acute respiratory infections in Zarqa, Jordan, during March–April 2012 (*1*). New cases and clusters of MERS-CoV infections continue to occur within the Arabian Peninsula, and the virus has been exported to other countries around the world.

For 2 persons affected by the April 2012 outbreak, the cause of death remained unknown until late 2012, when retained samples produced positive MERS-CoV results according to reverse transcription PCR. In May 2013, we obtained serologic and epidemiologic data from 124 persons: the 2012 outbreak survivors, their exposed contacts, and their household members. In that investigation, we found another 7 persons with positive MERS-CoV results according to ELISA and confirmatory results by immunofluorescence assay (IFA), microneutralization assay, or both (*1*). Results were indeterminate for another 8 exposed persons, whose results were positive by only 1 of these serologic methods; these 8 persons were deemed MERS test–negative overall.

For patients with severe acute respiratory syndrome coronavirus (SARS-CoV) infection, antibodies persist for at least 2 years after symptomatic infection (*2*). Recently, antibodies against MERS-CoV were found (by ELISA and IFA) in 9 healthcare workers in Saudi Arabia with symptomatic MERS-CoV infection at least 18 months after infection (*3*). Duration of antibody responses beyond 18 months has not been reported (*4*). Our objective was to evaluate long-term antibody responses among persons with laboratory-confirmed to MERS-CoV infection. 

## The Study

All surviving members of the 2012 outbreak in Jordan, their exposed contacts, and their household members who were identified serologically as either MERS-CoV positive or indeterminate were asked to consent to further participation. Participants were asked to provide a follow-up serologic specimen so we could compare 34-month results with 13-month results.

Specimens were prepared by the Jordan Central Public Health Laboratory (Amman, Jordan) and tested at the US Centers for Disease Control and Prevention (Atlanta, GA, USA). Antibody titers in serum samples were determined by an anti–MERS-CoV nucleocapsid indirect ELISA and by MERS-CoV (Hu/Jordan-N3/2012 strain) indirect IFA (*1*). The presence of neutralizing antibody titers was determined by microneutralization with live MERS-CoV (Hu/Jordan-N3/2012 strain) in a Biosafety Level 3 laboratory as described previously (*1*). Neutralization titers were defined as the reciprocal of the highest serum dilution completely protecting the Vero cell monolayer from cytopathic effect in at least 1 of 3 parallel wells. Titers >1:20 were reported as positive.

Of the 15 surviving persons with >1 positive serologic test result, 13 (87%) consented to follow-up testing. All 7 (100%) surviving persons with >2 positive serologic test results 13 months after the MERS-CoV outbreak also consented. Each of these 7 persons was considered to be a probable MERS-CoV case-patient according to World Health Organization criteria; each had had a symptomatic acute respiratory infection during the outbreak period and documented, unprotected exposure to >1 person with a case confirmed by reverse transcription PCR. 

For the 7 probable case-patients, ELISA titers at 34 months ranged from <400 to 1,600, representing reduced antibody titers compared with the 13-month estimates (400–6,400) for all but 1 person (Table). A nurse who worked in an intensive care unit (participant 06) and cared for confirmed case-patients during the outbreak was the only participant for whom ELISA indicated a consistent titer of 1,600 at both times.

**Table:** Characteristics of Middle East respiratory syndrome patients and antibody titers at 13 and 34 months after 2012 outbreak, Jordan*

Patient no.	Age, y/sex	Underlying condition	Intensive care	Days hospitalized	Chest radiograph†	13-mo titer‡				34-mo titer‡		
						ELISA	IFA	MN		ELISA	IFA	MN
02	31/M	Atrial septal defect	Yes	16	Right lobar pneumonia	>6,400	Pos	160		1,600	Pos	80
03§	60/M	Hypertension	NA	NA	Bilateral consolidation; pneumonia	400	Pos	20		<400	Ind	20
04	35/M	Hypertension	Yes	8	Bilateral lobar pneumonia	>6,400	Pos	80		400	Pos	40
06	46/M	None reported	No	6	Right bronchial congestion with bronchovascular markings	1,600	Pos	20		1,600	Pos	20
09	45/M	None reported	No	10	Left consolidation; right infiltrate	400	Pos	40		<400	Neg	40
11¶	41/F	None reported	No	4	ND	1,600	Pos	<20		400	Neg	<20
HH303§	39/F	Pregnant	NA	NA	NA	1,600	Pos	80		400	Pos	80

*IFA, immunofluorescence assay; Ind, indeterminate; MN, microneutralization assay; NA, not applicable; ND, not documented; neg, negative; pos, positive. †Taken within 3 days of presentation.  ‡Antibodies against Middle East respiratory syndrome coronavirus. §Symptomatic but refused hospitalization.  ¶Classified overall as being serologically negative at 34 mo.

Of these 7 participants, 6 (86%) had neutralizing antibody titers ranging from 20 to 80 at the 34-month follow-up evaluation, and only 2 (29%) had any decrease in neutralizing antibody titers over time. One participant had no detectable neutralizing antibodies (Figure). Of the 7 participants for whom IFA results were positive at 13 months, 4 (57%) had positive results at 34 months. 

**Figure:**
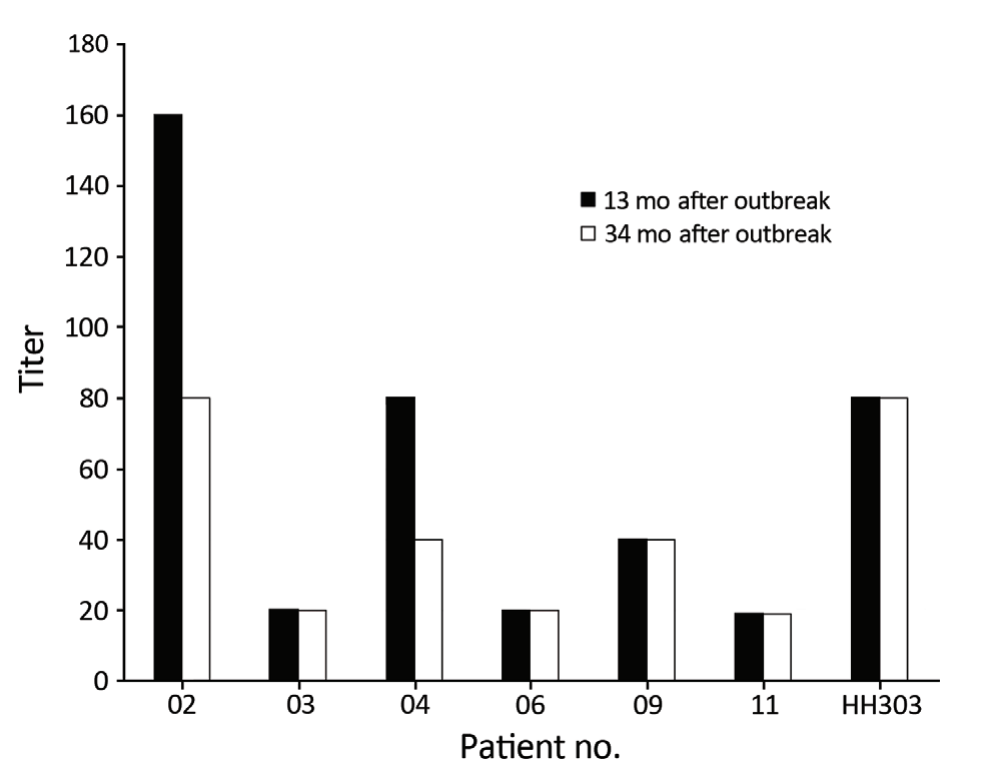
Neutralizing antibody titers against Middle East respiratory syndrome coronavirus (Hu/Jordan-N3/2012 strain) among 7 surviving case-patients at 13 and 34 months after the 2012 outbreak in Jordan. Patient numbers match those in the Table.

For the 8 surviving participants whose serologic results at 13 months were indeterminate, 6 (75%) consented to further testing. Of these, 3 (50%) reported having had no respiratory symptoms, 1 reported having had mild respiratory symptoms, and 2 had been hospitalized with respiratory infections; all 6 had had documented, unprotected exposure to >1 case-patient. Each of these 6 persons had negative serologic test results at 34 months and continue to be considered negative overall.

## Conclusions

Antibodies against MERS-CoV, including neutralizing antibodies, persisted in 6 (86%) of 7 persons 34 months after the 2012 MERS-CoV outbreak in Jordan. The observed persistence of these antibodies contributes to the understanding of individual immune responses to MERS-CoV infection, of population-based immunity in regions where MERS-CoV outbreaks have occurred, and to efforts for developing effective vaccines and therapeutics to counter MERS-CoV infections. 

Notwithstanding improvements in public health awareness and infection control practices in affected countries on the Arabian Peninsula and in the Middle East, emergence of the virus (e.g., its introduction to South Korea and the resultant epidemic of 2015 [*5*]) ongoing. MERS-CoV continues to pose grave risks to international healthcare and socioeconomic systems (*6*).

It has been hypothesized that mild or asymptomatic MERS-CoV infections are potentially associated with lower levels of MERS-CoV neutralizing antibodies over time (*7*). All 7 case-patients reported here had respiratory symptoms, were relatively young, and had few underlying medical conditions (Table). Any association between our MERS-CoV antibody results and clinical severity is therefore difficult to assess. Nonetheless, of the 5 persons for whom chest radiographs showed substantial changes within 3 days of symptom onset, each remained positive by microneutralization (>20) 34 months after the outbreak.

Although some similarities in the short-term development of antibodies against MERS-CoV and SARS-CoV (e.g., seroconversion 2–3 weeks after illness onset) have been observed (*8*,*9*), longer term serum antibody kinetics of these infections have not yet been compared. After SARS-CoV infection, robust IgG titers were observed through the second year but declined substantially during the third year after infection (*10*). Our finding of generally reduced but persistent MERS-CoV antibody responses even at 34 months suggests the potential for longer lasting antibody-mediated protective immunity against reinfection. However, whether such long-lasting antibodies can prevent reinfection or affect clinical outcome has yet to be examined. Diverse individual antibody test results allude to a potential role of genetic factors in explaining observed differences in immunologic responses to MERS-CoV exposure and infection. 

The times at which MERS-CoV antibodies were measured in our study were chosen because of logistics and field practicalities. Although limited outbreaks of MERS-CoV have occurred in Jordan since 2012, contact tracing efforts by investigators in Jordan lead us to believe that these persons were not subsequently exposed. The observed ELISA titers and neutralizing antibody titers support this supposition; otherwise, we would expect increases resulting from a booster effect after secondary exposure and infection. To further assess the duration and resiliency of MERS-CoV antibodies in human populations, continued follow-up serologic evaluations of these persons would be desirable. 
